# General statistical model shows that macroevolutionary patterns and processes are consistent with Darwinian gradualism

**DOI:** 10.1038/s41467-022-28595-z

**Published:** 2022-03-02

**Authors:** Mark Pagel, Ciara O’Donovan, Andrew Meade

**Affiliations:** School of Biological Sciences, University of Reading, Reading, RG6 6UR UK

**Keywords:** Evolutionary theory, Phylogenetics, Zoology

## Abstract

Macroevolution posed difficulties for Darwin and later theorists because species’ phenotypes frequently change abruptly, or experience long periods of stasis, both counter to the theory of incremental change or gradualism. We introduce a statistical model that accommodates this uneven evolutionary landscape by estimating two kinds of historical change: directional changes that shift the mean phenotype along the branches of a phylogenetic tree, and evolvability changes that alter a clade’s ability to explore its trait-space. In mammals, we find that both processes make substantial independent contributions to explaining macroevolution, and are rarely linked. ‘Watershed’ moments of increased evolvability greatly outnumber reductions in evolutionary potentials, and large or abrupt phenotypic shifts are explicable statistically as biased random walks, allowing macroevolutionary theory to engage with the language and concepts of gradualist microevolution. Our findings recast macroevolutionary phenomena, illustrating the necessity of accounting for a variety of evolutionary processes simultaneously.

## Abstract (web summary)

‘Macroevolution posed difficulties for Darwin and later theorists because species frequently change abruptly, or experience long periods of stasis, both counter to the theory of incremental change or gradualism. Here, the authors propose a macroevolutionary statistical model that accommodates this uneven evolutionary landscape, and shows how even abrupt macroevolutionary changes are compatible with gradualist microevolutionary processes.’

## Introduction

Statistical models of macroevolution attempt to describe the historical phenotypic changes that gave rise to the major differences observed among species. Three widely used classes of such models—Brownian motion, pulsed change or jumps models, and models of adaptive landscapes—differ in how they characterise these changes on phylogenetic trees. Brownian-motion^1,2^ models, including early burst^3,4^ and variable-rates^5–9^ approaches, presume that phenotypes follow adaptive optima that wander incrementally and continuously according to an unbiased random walk, mathematically equivalent to neutral drift. The key element of the Brownian process is the variance of its incremental steps per unit of time (hereafter the *Brownian variance*): denoted by *σ*^2^, it is a measure of ‘evolvability’^10^—the capacity of an evolving system to explore its trait-space. Early burst models fit a trajectory of increased *σ*^2^ early on, followed by a gradual return to the background variance. This allows for the possibility that phenotypic divergence might have been enhanced early in the history of a tree. Variable-rates models allow the Brownian variance to change ‘non-parametrically’, that is, without imposing any trajectory of change on the tree, or constraints on the timing or location of changes.

Models of ‘pulsed’ change depart from the incremental wandering of Brownian models. They seek to characterise the large and often abrupt phenotypic ‘jumps’ such as Simpson^11^ and later Gould and Eldredge noted^12^, and that fall probabilistically beyond the range of the neutral Brownian variance. They include the ‘stable’ distribution^13^ and Levy process^14^ methods. The former accommodates large changes directly from the stable distribution, which can produce wider or fatter tails (bigger jumps) than are expected under neutral Brownian motion; Levy process models characterise large changes from a separate jumps process that acts in addition to the Brownian variance. In both cases, evolvability is effectively increased along a branch of the phylogeny—or successive branches—to explain the rapid change.

The popular (Cooper et al.^15^, their Fig. 1) ‘adaptive landscape’ models^16–19^ suppose that stabilizing selection towards local or general optima dominates macroevolution. Landscape models envision that a trait *X* evolves under random diffusion and deterministic forces. Diffusion is modelled as an unbiased Brownian-motion random walk, while the deterministic component imposes a force that draws the trait towards an optimal value. Adaptive landscape models include the Ornstein-Uhlenbeck (OU) and Fokker–Planck–Kolmogorov (FPK) approaches of which the OU is a special case. The deterministic component of the FPK model is flexible^17^, allowing complex landscapes and a variety of approach-trajectories towards presumed optima. The single-optimum OU model can also be modified to fit more than one adaptive optimum^16,18,20–22^ among the lineages of the phylogenetic tree.

**Fig. 1:**
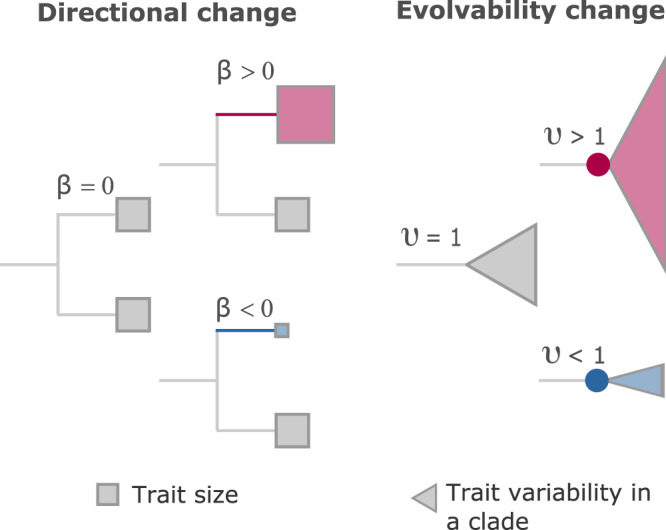
Description of Fabric model effects. Directional changes occur along branches and increase (*β* > 0, red), decrease (*β* < 0, blue) or leave the phenotype unchanged (*β* = 0). They affect all downstream descendants (squares). Directional changes do not alter evolvability. The directional effect along a branch is the product of *β* and the branch length, *t*, meaning that *β* is measured in units of instantaneous change and $\beta \times t$ records the phenotypic change along a branch. Evolvability changes occur at ancestral nodes (circles) and multiply the Brownian variance *σ*^2^ by a quantity *υ*. They can increase (*υ* > 1, red), decrease (*υ* < 1, blue) or leave the background variance unchanged (*υ* = 1). Evolvability effects do not change the mean phenotype within a clade. Both parameters’ magnitudes can vary throughout the tree. Combinations of directional and evolvability effects can occur. For example, a positive directional change could be followed by a decrease to the Brownian variance (*υ* < 1), indicating that the move towards increasing the mean phenotype was followed by a period of reduced evolutionary exploration (possibly indicative of a narrow niche-space).

The drift, directional jumps and landscape models characterise a wide range of phenomena, but their parametric specialisations mean that they will normally give differing views on the nature of macroevolution, even when applied to the same data: Brownian motion models will tend to find drift, early bursts, and other changes to *σ*^2^, abrupt-change models will tend to find extra-Brownian jumps and landscape models will often find evidence for what looks like stabilising selection when each of these processes, and others, may all be at work in the same data.

The model we introduce and study here—called the Fabric model—separates directional changes to phenotypes from evolvability changes (i.e., changes to *σ*^2^), and finds instances throughout a phylogenetic tree of their occurrences against a background of neutral Brownian motion (Fig. 1, Methods). Where the existing macroevolutionary models suppose that the Brownian variance, *σ*^2^, must be increased or augmented to accommodate large directional changes, it is reasonable to expect that large or abrupt phenotypic shifts do not necessarily signal or require changes to evolvability. Instead, our approach allows any links that might occur between directional phenotypic effects and changes to evolvability to emerge empirically throughout the phylogenetic tree. Either alone or in combination, these changes can describe a variety of differing macroevolutionary patterns, or what we call the fabric of macroevolution.

The model identifies a directional phenotypic change by the parameter *β* (Fig. 1, Methods). Directional changes occur along a branch and shift the phenotype of the trait being investigated by an amount *β* × *t*, where *t* is the length of the branch in which they occur, affecting all descendant species. The size of *β* parameters varies among branches. Regardless of the amount of phenotypic change they bring about, directional changes do not alter the Brownian variance, *σ*^2^. The Fabric model does not include or require any special jump mechanism, nor does it link the directional phenotypic changes to increased variance. Instead, we suppose—and show how to test empirically—that the *β* × *t* effects can be characterised as biased random walks, making use of the incremental changes drawn from the Brownian variance. This allows the macroevolutionary directional effects to be understood as extensions of well-known gradualist microevolutionary processes that describe changes within populations from one generation to the next. To the extent that the model can even accommodate abrupt directional changes this way, longstanding debates about the mechanisms that produce them—such as those raised in the context of ‘punctuated’ change^12^—fade away.

‘Evolvability’ changes, denoted by *υ*, occur at nodes of the tree and *increase* or *decrease* the Brownian variance of the clade descending from that node according to *υσ*^2^; they alter the range of outcomes, but without changing the mean phenotype (Fig. 1, Methods). Like the *β*, their values can vary throughout the tree and include periods of reduced evolutionary exploration (*υ* < 1) such as might arise from a narrow niche-space, but also ‘watershed’ moments of probably enhanced evolvability (*υ* > 1) that lead to a wider than expected range of outcomes. Watershed moments might arise from a ‘key innovation’^23^, or from changes intrinsic to an organism (e.g., an increased genomic mutation rate) that amplify its descendant species’ evolutionary potentials^10^.

The species’ data in combination with a phylogeny determine the placement on the phylogeny of directional (*β*) and evolvability (*υ*) changes, their magnitudes, and any empirical links between them. Our methodology does not constrain the number, position or trajectories of effects, but requires them to ‘pay’ their way in a Markov chain Monte Carlo setting by improving the fit of the model to the data.

By comparison to parametric models that fit one or a small number of processes throughout a tree, the Fabric model is typically rich in parameters, and this requires care in fitting and interpretation (see Methods, Selection of parameters; Supplementary Methods, Identifiability, Supplementary Fig. 2a, b). But the Fabric model’s approach avoids the potential for one or a handful of effects in a tree to give the impression that the parametric model’s form is true everywhere in the tree when it might not even be true of any part of the tree.

We apply the Fabric model here to the evolution of body size in mammals, a Class particularly suitable for macroevolutionary study owing to its wide morphological variation and ecological specialisations. We use logarithmically transformed (base 10) data on *n* = 2859 mammalian species’ sizes^24,25^, arrayed on the Mammalian clade of the TimeTree: Timescale of Life^26^, which includes Monotremes and the Marsupials. Our interest is to use the mammals to explore what the model reveals about the patterns of macroevolution, and to try to advance our understanding of macroevolutionary phenomena by linking them to well-understood microevolutionary processes. We show how to test long-standing hypotheses about the nature and timing of macroevolutionary events and our results recast some macroevolutionary trends.

## Results

We studied five versions of the model allowing each to run to stationarity in a Markov chain that explored its particular set of parameters (Methods, Eqs. (1)–(4)): a model of Brownian motion to capture the process of unconstrained and incremental evolution (i.e., all *β* = 0 and all *υ* = 1); a ‘directional model’ that included a vector of inferred directional phenotypic change parameters ***β*** (bold highlighting refers to a vector of individual effects) in addition to Brownian motion, but all *υ* = 1; an ‘evolvability model’ including a vector of inferred multipliers ***υ*** that alter the variance of Brownian evolution, but with all *β* = 0; a ‘combined model’ that includes both directional and evolvability effects (both the *β* and the *υ* are allowed to vary), and a combined model that also includes a global directional trend parameter *β*_*g*_ (Methods, Eq. (5)). Here, *β*_*g*_ assesses evidence for a general trend towards either larger or smaller body size from the ancestor of all mammals at the root of the tree to the contemporary species in lineages that have experienced differing average evolvability. The parameter takes advantage of the fact that increased evolvability (*υ* > 1) is mathematically equivalent in a Brownian evolution model to evolving for a longer period of time at the background Brownian variance, *σ*^2^, and vice versa for *υ* < 1.

We compare the models using their marginal likelihoods as calculated from the ‘stepping-stones’ method^27^ (Methods). Marginal likelihoods calculated this way approximate the integral of the likelihood over the entire range of a model’s priors on each of its parameters, and so naturally penalise models with more parameters. The ratio of two marginal likelihoods (the difference of their log-marginal likelihoods) can therefore be treated as a *BayesFactor*.

### Directional and evolvability changes are substantial and distinct

Comparison of the five models’ marginal likelihoods (Table 1) illustrates one of the central points of this article: that a statistical description of macroevolution must account for the substantial and distinct contributions of directional (*β*) and evolvability (*υ*) changes: modelling one of these processes at the expense of the other or linking them a priori, risks missing important elements of the macroevolutionary picture. The two processes on their own each improve the marginal likelihood over the Brownian model by a similar amount. Then, the combined model improves the marginal likelihood by roughly twice that of either the directional or evolvability models alone, and with very little change to the numbers of directional and evolvability parameters, indicating that these parameters respond to different historical signals. Simulation studies (Supplementary Tables 1 and 2, Supplementary Fig. 1) confirm that the models are identifiable, the method estimates its parameters well and discriminates between the directional and evolvability changes.

**Table 1:** Models and description of outcomes.

Model	Marginal log-likelihood ± sd^a^	Brownian variance^b^	Ancestral mass *α*, g (95% CI)	No. of β parameters	No. of υ parameters	Δ likelihood^c^
**Brownian**	−922 ± 0.02	0.0088 ± 0.0002	776 (707–851)	–	–	–
**Brownian** **+** **β, (υ** **=** **0)** (directional model)	−709 ± 5.19	0.0040 ± 0.0002	335 (37–2980)	415	–	+213
**Brownian** **+** **υ, (β** **=** **0)** (evolvability model)	−654 ± 6.79	0.0038 ± 0.0007	162 (2.2–1176)	–	137	+268
**Brownian** **+** **β** **+** **υ** (combined model)	−565 ± 5.00	0.0031 ± 0.0003	332 (108–1002)	413	125	+357
**Brownian** **+** **β** **+** **υ** **+** **global β**^d^ (combined model with global trend)	−562 ± 4.11	0.0036 ± 0.0018	32.4 (29.5–34.7)	417	119	+360

^a^See Methods for the description of marginal likelihoods; standard deviation based on six independent chains.

^b^The smaller estimate of the ancestral body size and smaller Brownian variance of the model that only includes evolvability effects are artefacts arising from the scaled tree (Methods, “Model”). Most evolvability effects are >1 (text), and a longer total tree length implies a smaller Brownian variance; this artefact does not apply to the directional model.

^c^Difference in log-marginal likelihoods compared to Brownian motion. Marginal likelihoods tend to penalise models with greater numbers of parameters, such that any numerical difference between two models is taken as evidence of a better model.

^d^Global *β*g = 0.0056 ± 0.0026 (see text); in a model without branch-wise directional effects, *β*g = 0.010 ± 0.0015.

The directional model finds a smaller body size for the ancestor of present-day mammals and estimates the Brownian variance (*σ*^2^) to be roughly half of that in the Brownian motion model (Table 1; all parameter estimates we report are means of the *n* = 1000 posterior samples from a stationary Markov chain, having adjusted the sample size for autocorrelation; Methods). The Brownian motion model, lacking any explicit mechanism to produce directional change, requires a large variance to accommodate the observed phenotypic differences among species. By comparison, the directional model can accommodate these same phenotypic differences with a lower background Brownian variance by understanding them as the outcome of biased random walks (see below). This has implications for quantitative macroevolutionary theory and for estimating ancestral states. The smaller estimate of the ancestral body size and smaller Brownian variance of the evolvability model are artefacts of the altered phylogenetic path lengths in that model (see Methods, “Model”).

The marginal likelihoods (Table 1) moderately support the combined model (i.e., directional and evolvability effects) with the global directional trend and we use that model in all further analyses. The global trend further reduces the inferred size of the 172 million-year-old ancestral mammal (Table 1): the value of ~32.4 g compares to the estimated size of *Eomaia scansoria*, a Eutherian (placental) fossil which existed ~120 million years ago and is believed to have weighed ~25 g^28^. The slope of the global trend at 0.0056 ± 0.0026 (95% CI = 0.0025–0.0120) is best understood when expressed as 10^0.0056^ = 1.013*-fold* increase in un-logged size (Eq. (1); changes along branches are ratios on the un-logged scale) per million years of evolution (Methods). Being positive, this global trend accords with the phenomenon known as Cope’s rule^29–31^. But consistent with the marginal likelihoods, the effect is small, accounting for, on average, an increase in the size of around 376 g from the estimated ancestral mammal to the extant species, or roughly 2 g per million years throughout the 172 million years of mammalian evolution the tree represents.

**Fig. 2:**
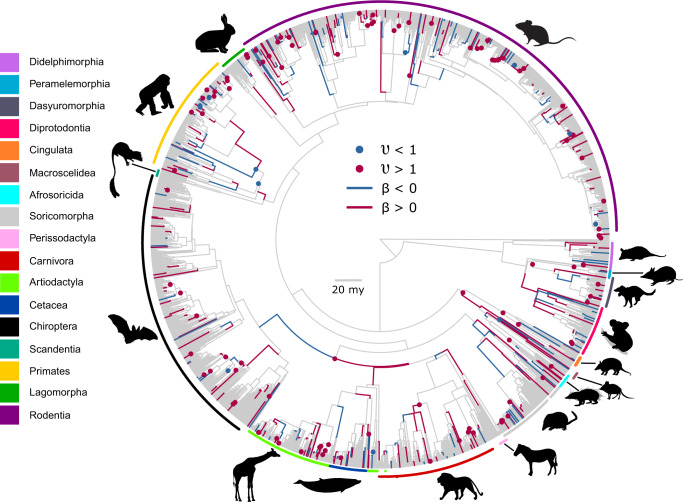
The tree of the mammals used in this study (see text), showing the widely scattered positions of the 417 directional and 119 evolvability effects. The legend identifies the Order or in some cases sub-order corresponding to the colours around the perimeter of the tree. The largest positive directional effect in the tree, at $\beta \times t=1.98$ and corresponding to a near 100-fold increase in size along a 7.6 million-year branch, identifies ten baleen whales (Cetacea) whose sister group are the smaller dolphins and porpoises. The largest negative directional effect at −2.11, a >100-fold reduction in size along a branch of 2.25 million years, identifies a group of forty elephant shrews (Macroscelidea), separated by 5 million years from their sister taxa the Aardvarks. A $\beta \times t=-1.29$ signifying a 20-fold reduction in size describes the descent from the remaining Laurastheria Orders (including Carnivora, Cetaceans, Perissodactyls and the Artiodactyla) to the ancestral bat. The largest increase to evolvability at $\upsilon =8.16$, corresponding to an eight-fold increase in the Brownian variance, identifies a clade of nine Dasyurid marsupials which range in size from 212 g to 8.2 kg. In keeping with the size of *υ*, the across-species phenotypic variance of sizes is this group is approximately nine times greater than the Brownian variance. The greatest reduction to evolvability at 0.014, corresponding to a seventy-fold reduction in the variance, identifies a clade of five Peromyscus rodents who all weigh within one gram of each other. The across-species phenotypic variance among the five small rodents is ~1/72^th^ that of the Brownian variance, while other species in the same genus, separated by just 380,000 years of evolution, range in size from 14 g to 71 g. All silhouettes are downloaded from www.phylopic.org. In the order of the legend: silhouettes 1, 3 and 4 are attributed to Sarah Werning and available for reuse under the Creative Commons Attribution 3.0 Unreported license https://creativecommons.org/licenses/by/3.0/. Silhouette 12 is attributed to Chris huh [sic] and available for reuse under the Creative Commons Attribution 3.0 Unreported license https://creativecommons.org/licenses/by/3.0/.

The combined model identified 417 directional and 119 evolvability changes (Table 1, Methods, *Selection of parameters*), widely scattered throughout the Mammalian tree (Fig. 2; we do not further investigate their phylogenetic distribution here). These historical effects translate to contemporary signatures of directional evolution residing in 93.3% of the species and of changes to evolvability in ~66.6% of contemporary species. The average correlation in the posterior sample between the observed and predicted data at the tips of the tree (obtainable from Eqs. (4) or (5)) is 0.90 ± 0.03, 95% range 0.82-0.95; we do not explore the predictive capabilities of the model further in this paper.

Consistent with the marginal likelihoods, evidence for links between directional and evolvability changes is negligible. At the level of individual branches in the tree, just eight (~6.7%) out of a possible 119 pairs of directional and evolvability changes that could occur in the same branch, co-occur in the posterior sample significantly more often than expected by chance. Of these eight, two (~1.7%) record instances in which a directional shift is paired with a reduction in the Brownian variance i.e., $(\upsilon\, < \,1)$, while in the remaining six cases a directional shift is paired with an increased exploration of the trait-space i.e., $(\upsilon\, > \,1)$.

The number of directional phenotypic shifts along a path leading from the root of the tree to a species is not correlated with the number of evolvability changes that occur along that same path (Poisson regression, *p* = 0.186 against an intercept-only model including phylogeny). The numbers of evolvability and directional changes along a path both significantly and independently correlate with body size: numbers of positive directional shifts and increases to evolvability along a path correlate with increased size (*p*-values < 0.001), and negative directional changes and reduced evolvability correlate with smaller size (*p*-values < 0.001); but interactions between numbers of directional and evolvability changes are not significant.

These results suggest two pathways in the mammals to long-term changes in size: one in which increases in size occur additively with greater evolutionary exploration and one in which becoming smaller occurs with reduced evolutionary exploration (as measured by reduced *σ*^2^). Why the latter relationship arises we cannot say but could reveal something fundamental about the nature of the evolutionary size-niches available to mammals.

### Directional changes are large and possibly abrupt

**Fig. 3:**
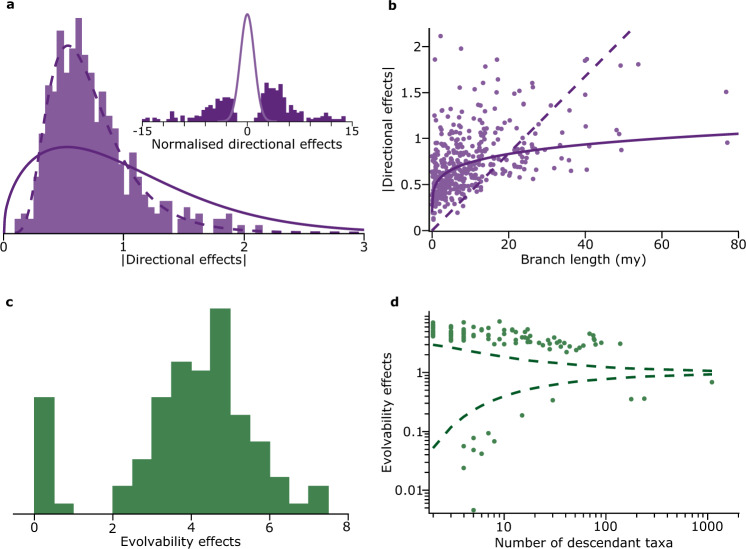
Directional and evolvability effects. **a** Absolute values of the mean branch-wise $(\beta \times t)$ directional effects (mean = 0.73 ± 0.34, *n* = 417, 5.37-fold average change): solid curve is the Bayesian prior, dashed curve is a best-fitting lognormal distribution. Inset: Normalised directional effects fall outside the standard normal curve expected under Brownian motion. More positive than negative *β*s (247 vs. 170, *p* = 0.00169, two-tailed binomial test); **b** dashed line: best-fitting regression line of steady change, constrained to go through origin; solid line: best-fitting curve linking ${{\log }}_{e}\left(\beta \times t\right)$ to ${{\log }}_{e}t$. The curve shows that the size of the directional effect is only weakly related to time in the branch, consistent with directional effects occurring in bursts; **c** the distribution of median evolvability effects (median = 4.23, interquartile range 3.19–4.94): increases in evolvability outnumber decreases ~8:1; **d** evolvability effects fall outside the 5th and 95th percentile boundaries (dashed curves) derived from the probability density of *υ* (Methods, Eq. (7)), and converge on 1 as the number of taxa affected increases (Methods). Source data are provided as a Source Data file.

The distribution of the absolute values of the *n* = 417 branch-wise directional effects (Fig. 3a) has a mean = 0.73 ± 0.34 per branch (we report the directional changes as branch-wise effects = *β* × *t*, where *t* is the length of the branch in which the effect occurs). Because the data are logarithmically transformed a change of this magnitude indicates that descendant species typically diverge from their ancestors five-fold or more (10^0.73^ = 5.37) and occasionally 100-fold (*β* × *t* = 2, Fig. 1) along the branches of the phylogeny. These individual directional effects are the architects of body-size change in the mammals, being large by comparison to the weak global trend: the geometric mean directional change is 0.13 per million years (95% CI = 0.12–0.15, median = 0.109), roughly twenty times that of the global directional change parameter. Positive and negative directional effects do not differ in their average size (*p* = 0.96), but most directional effects (59%, Fig. 3a, inset, *p* < 0.0001) are towards increased size, perhaps reflecting more opportunities for the ancestrally small mammals to fill what was for them a mostly unexplored mammal size-space^30^.

A central question of macroevolution is whether directional phenotypic changes occur at a steady or regular pace, or are episodic, occurring in ‘bursts’ over shorter time periods that might be followed by longer periods of very little change or even stasis. In the present context, a regular pace of change throughout the period represented by a branch will yield *β* × *t* values in Fig. 3b that increase linearly with the length of the branch in which a directional change occurs (dotted line, Fig. 3b). Instead, we find that these changes are only weakly linked to the length of their branches (solid curve, Fig. 3b): some of the smallest and some of the largest directional changes occur in the shortest branches, but after around 10 million years the average change along a branch increases only negligibly. It could be that changes do accumulate steadily in each branch but at different paces dictated by their particular value of *β*, although this explanation struggles to understand the levelling off after around 10 million years. An alternative possibility is that directional changes tend to occur at or near the time of speciation, and then the phenotype remains relatively unchanged throughout the rest of the time period represented by *t*, as suggested by previous authors^14,30,32^. Whether there are multiple bursts or episodes of change in a branch or just a single event is beyond the resolution of most comparative data, but what can be said is the net amount of directional change in a branch appears not to be limited in any general way by the length of that branch.

Perhaps phenotypic change accumulates steadily but phylogenetic branches are poor measures of the relevant time—branches in phylogenies of extant species almost certainly conceal many past speciation events of now long-since extinct species. Were these speciation events present they would sub-divide the phylogenetic branches into shorter lengths. Change might have been steady in some of these, whilst remaining static in the others. We cannot rule out the possibility that the changes we observe in Fig. 3b occur steadily over shorter periods of time, but think it is unlikely: we find a strikingly similar pattern of change in a dataset of ancestral-descendant pairs of fossil mammals^29^, where the periods of time separating ancestors and descendants are roughly 1/10^th^ of the average branch length on the mammalian tree (Supplementary Fig. 3a, b). This concordance with the fossil data also reduces—in this case—the worry that comparative trends derived from contemporary species might only reflect the evolutionary processes that occurred in surviving lineages^30,33^.

### Directional phenotypic shifts do not require increased evolvability

**Fig. 4:**
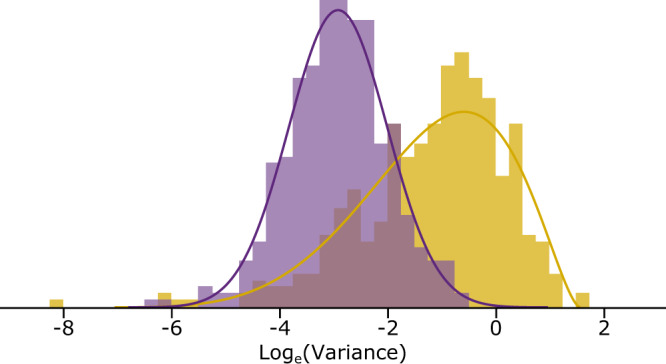
Inferred-phenotypic versus expected-mutational variances of change. The inferred-phenotypic variances of directional change along the branches corresponding to the *n* = 417 directional effects (purple histogram) are generally smaller than an estimate of the mutational variance that would be available along the same branches (mustard histogram; Supplementary Table 3). Sufficient mutational variance exists to produce the observed directional changes without recourse to increases in the variance of evolution, or to special jumps processes or fat-tailed distributions (see text). Source data are provided as a Source Data file.

We observe that the magnitude of phenotypic changes is not linked to the length of the branch in which they occur, but what does this imply about their mode of change? To accommodate large and abrupt phenotypic changes the variable-rates, fat-tailed and Levy jumps models propose either an increased variance or a special jumps process that adds its own component of variance on top of the Brownian variance. But these mechanisms potentially separate macroevolution from ordinary gradualist or incremental evolution and are not necessary. The *n* = 417 directional changes imply a difference in the value of the evolving trait (log_10_mass) between the beginning and end of the branch in which the directional change is observed. This difference can be expressed as a variance (Supplementary Eq. 2). Doing so, we find that these directional-change variances are generally smaller than a conservative estimate of the mutational variance^34,35^ that would be available along the same branches (Fig. 4, Supplementary Fig. 4, Supplementary Table 3).

The significance of the results in Fig. 4 is that new mutational variance generated within populations each generation is sufficient to sustain the directional shifts we observe in the mammals: they are explicable within the model as biased random walks where the incremental steps of evolution are drawn from the constant background Brownian variance; no special evolutionary mechanism is required. Directional selection can easily produce changes of the magnitudes we observe^36^, and it has long been recognised that the low overall rate of evolution in the mammals is often even compatible with neutral drift^35,37^; Fig. 4 shows these explanations could also be true of the over four-hundred instances of large phenotypic changes our model infers to have occurred throughout the history of the mammals. Figure 4 is an important result for macroevolutionary statistical models as it places the so-called abrupt or large phenotypic shifts in a gradualist or incremental framework and frees macroevolutionary theory to engage directly with the concepts and language of microevolution and population genetics.

### Enhanced exploratory potentials dominate changes to evolvability

Where changes to evolvability do occur in the mammals, ‘watershed’ moments of increased evolvability or exploratory potential dominate departures from the constant-variance Brownian wandering: enhanced evolvability (*υ* > 1) occurs repeatedly and independently throughout the tree (Fig. 2), and by a margin of ~8/1 over instances of *υ* < 1 (Fig. 3c; *p* < 0.000001, two-tailed), despite the cost the Bayesian prior imposes on these parameters being roughly symmetrical about its mode of 1, the default value. This reflects numerous and phylogenetically widespread increases in the potential for clades to explore the ecological and/or phenotypic width of trait-spaces relative to the background Brownian variance. Bouts of enhanced evolvability seem especially likely in small groups of closely related species, and they occur in the tree right up to the present (Fig. 2; changes to evolvability affecting fewer than ~10 taxa can be difficult to estimate, Methods, Eq. (7) and Supplementary Table 2, but our selection procedures guard against false positives).

Could changes to evolvability—being applied to entire clades of descendant species—merely summarise in a single parameter a set of contemporaneous directional changes? For example, a set of independent and diverging phenotypic size changes—that is, some positive and some negative—in the descendants of a common ancestor might appear to the model as a single change to evolvability, with *υ* > 1. We cannot be sure, but Table 1 suggests that directional effects (i.e., the *β* × *t*) on their own do not fill the evolvability parameter-space, and simulations (Supplementary Tables 1 and 2) suggest the same. We speculate that evolvability changes are detecting instances in which some characteristic of the common ancestor, something about the environmental niche, or both leads either to a more or less divergent ‘downstream’ pattern of evolution, possibly shepherded by selection. Their appearance can act as spurs to further hypothesis testing, such as proposed links between genomic changes and speciation^38^.

All the evolvability changes (i.e., the *υ*, Fig. 3d) fall outside the 5% or 95% confidence intervals of their probability distribution (Methods, Eq. (7)), indicative of strong effects. As predicted from their probability distribution, the *υ* parameters broadly converge on their null value of 1 as the number of species that are affected increases—it is unlikely that whatever it is that gives rise to alterations to evolvability persists across larger numbers of species.

### Macroevolution has been uniformitarian in magnitude and rate

Much macroevolutionary speculation has centred around the existence of significant evolutionary epochs (such as the K-T extinction), of phenotypic trends being constrained by contingency^39,40^, of parametric trajectories of trait evolution or of long periods of stasis. We find little evidence for any of these in the evolution of mammals. In what follows we first describe trends in the magnitude of directional and evolvability effects throughout mammalian evolution, followed by trends in their rate of accumulation through time.

**Fig. 5:**
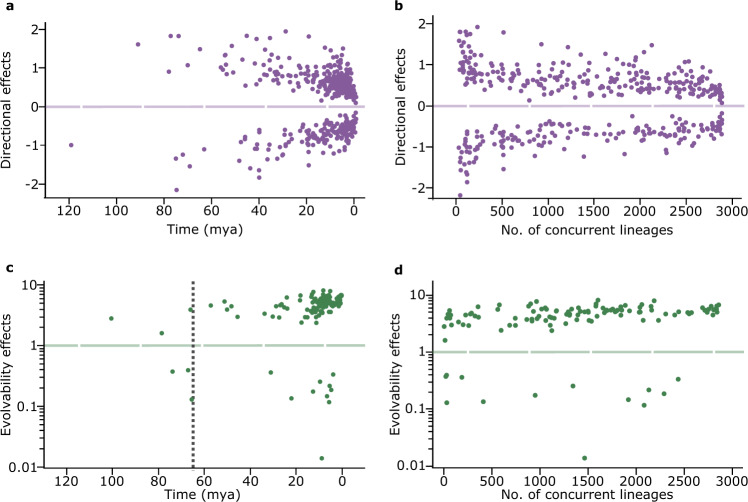
Directional and evolvability effects throughout mammalian evolution. **a** Mean directional changes plotted against time from the present (mya) show a slight tendency to be larger in the past; **b** Plotted against the number of concurrent lineages, the large-magnitude early changes are shown to be confined to a small number of lineages at the beginning of the tree; smaller recent changes concentrated in very late speciation events; **c** Distribution of events of enhanced and reduced evolvability is relatively unchanging across the tree with dotted, vertical line marking the K-T boundary 66 million years ago; **d** Same distribution plotted against the number of concurrent lineages. Source data are provided as a Source Data file.

Directional changes—both positive and negative—are larger in absolute value early in mammalian evolution (Fig. 5a)—perhaps coinciding with the diversification of the mammalian orders—and then smaller recently. But, the effect is small and when viewed through the lens of available lineages (Fig. 5b), these deviations in the average size of directional shifts are concentrated among a small number of historical lineages and then among a few species near the present. The remaining majority of changes paint a uniformitarian picture of similar average magnitudes and no tendencies to admit long periods during which little or no change occurs. Evolvability changes are roughly uniform in magnitude throughout the evolution of the mammals (Fig. 5c, d).

Could the temporally early and late deviations of the directional change parameters from average magnitudes of change (Fig. 5a) reflect limitations of the comparative method? Perhaps only the signatures of large deviations survive from the distant past, while closer to the present it remains possible to detect smaller changes. Fossil data from the mammals (Supplementary Fig. 3c) also show a tendency—although less pronounced than that in Fig. 5a—to be larger in the past. On the other hand, our simulation studies show that smaller changes are less likely to be detected in the distant past as these signals get erased by stochastic effects or by other systematic changes.

Previous work^41^ using comparisons among contemporary clades of animals has suggested that large phenotypic differences tend only to occur after those clades have been separated by a ‘million-year wait’, or more, as measured by twice the difference in time from their common ancestor. Figure 5a, b suggests an alternative interpretation: the directional changes that produced these contemporary differences could have occurred in the branches immediately after separation from their common ancestor. Unless multiple directional shifts occurred in parallel in later branches the changes associated with the ‘million-year wait’ might have occurred over far shorter time periods in the distant past, and were then retained for millions of years.

The temporal pattern of evolvability changes (Fig. 5c, d) can be used to examine a previous suggestion that mammalian evolution conformed to a single-optimum OU model up to the K-T extinction boundary (~66 mya), followed by incremental Brownian evolution^42^. We find three instances of enhanced evolvability early in the tree, followed by three later instances of reduced evolvability prior to the K-T extinction boundary (Fig. 5c). This is the opposite to the OU trajectory for a single optimum. That model expects the interspecific phenotypic variance to start out small and then increase to its equilibrium. And, only one of the three pre-K-T reductions in evolvability is nested within an earlier change (Supplementary Fig. 5), so these six changes are mostly unrelated, and not part of a general evolutionary trajectory whatever they might signify. We don’t take this as a test of the OU model or even of this earlier study’s more general assertion about evolution in that time period. Rather, we use it to illustrate the value of being able to visualise individual changes on a tree.

**Fig. 6:**
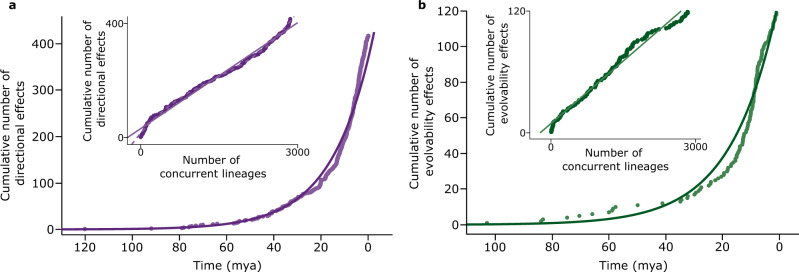
Rate of occurrence of directional and evolvability effects. **a** The number of events of directional change since the common ancestor of the mammals accumulate approximately exponentially with time (*r*^2^ = 0.99), reflecting the increase arising from speciation in number of concurrent lineages in the tree. Inset: accumulation of directional events is linear with the number of concurrent lineages: fit to solid straight line implies constant accumulation of effects as new lineages become available owing to speciation (*r*^2^ = 0.990, slope = 0.125 ± 0.0006 directional events per lineage). Cubic polynomial (dashed) is significant (*r*^2^ = 0.998) suggesting slightly higher rates early on and at the end, but the effect is small; **b** Evolvability events accumulate approximately exponentially in time (*r*^2^ = 0.94 for best-fitting least squares model). There is a suggestion of a slowing of evolvability changes between 10 and 20 mya. Inset: Straight line accumulation of evolvability changes as a function of the number of lineages implies constant rate of accumulation of effects as new lineages become available owing to speciation (*r*^2 ^= 0.98, slope = 0.041; ± 0.0005 per lineage). Time, no. of concurrent lineages, and their interaction all yield significant effects when jointly predicting either the cumulative number of directional effects (*R*^2 ^= 0.998) or evolvability effects (*R*^2 ^= 0.994), although with *R*^2^ this high, the partial contributions can be negligible and yet still attain significance. Source data are provided as a Source Data file.

Untangling temporal effects from the ‘opportunity’ for change to occur can help to clarify apparent trends, and test hypotheses about history. Numbers of directional and evolvability changes both accumulate approximately exponentially with time (Fig. 6a, b). But this reflects the exponential rise in the number of concurrent lineages in the tree: instances of both parameters accumulate linearly, that is, at a constant rate, with the number of those lineages: (0.125 ± 0.0006 per lineage for directional events, 0.041 ± 0.0005 per lineage for evolvability changes). If there is a moral here it is that species may not know what year it is but they are aware of the presence of other species.

Directional changes might have occurred more frequently early in mammalian evolution (Fig. 6a, inset, dashed line, cubic polynomial, *p* < 0.001)—possibly coinciding with the separation of the Mammalian Orders—before settling down to a roughly constant rate of accumulation, only to increase again nearer to the present. But any such effects are small, accounting for <1% of the variance. The rate of accumulation of changes to evolvability doesn’t show the obvious departures from linearity present in the directional shifts: opportunities for exploring the trait-space arise at a steady pace (Fig. 6b, inset). The steady accumulation of both kinds of changes suggests a uniformitarian picture of macroevolution, one of continually evolving opportunities in which neither significant evolutionary epochs nor historical contingency seems to play a large role. Once again, we observe a similar pattern in the fossil data (Supplementary Fig. 3d)

## Discussion

A sufficient account of macroevolution needs to distinguish and then separately consider both directional and evolvability changes, and their possible interactions, throughout the history of a group of species. Here, the two processes contribute roughly independently to the fit of the model to the data, suggesting that in mammals at least, size changes seem not to be linked, or only negligibly, to changes in evolvability. On their own, models of incremental Brownian evolution, fat-tailed models, models of evolutionary optima or simple early burst models potentially miss important phenomena or mistake them for each other. They may also, by fitting parametric trajectories of change, miss the detailed heterogeneity of macroevolution or allow a small number of events to sway them: events of directional change and shifting evolvability are scattered throughout the history of Mammalian evolution and, at least in our data, do not conform in any obvious ways to trends or parametric trajectories. This is not to say that other models should be ignored, but perhaps that they are best applied to specific circumstances that test an a priori set of hypotheses^43^, and even then compared to descriptions such as we provide here.

The directional shifts and evolvability changes we observe lend themselves to evolutionary hypothesis testing. The abrupt phenotypic changes observed in the fossil record and inferred on phylogenies are often seen as challenges to Darwinian gradualism, being attributed to occult forces such as quantum changes, macromutations and megaevolution^11^, or phenomenologically to special jumps processes embodied in fat-tailed distributions or Levy processes. But our results show that the directional changes we infer can arise from the ‘ordinary’ incremental mode of Darwinian evolution available to an existing genetic system, without requiring any alteration to that system’s ability to produce new mutational variants. This is an important result, providing a way for macroevolutionary theory to make progress by engaging directly with well-understood concepts and measurable phenomena that occur at the microevolutionary level.

The changes to evolvability we identified do not show the patterns expected from either an early burst or a single-optimum OU model. We find instances of reduced evolutionary exploration right up to the present where the single-optimum OU model expects the interspecific variance to have risen to its equilibrium. We also find many instances, again right up to the present, of independent ‘watershed’ moments in which a clade’s future evolutionary potential is amplified^10^—often five-fold or more. This increased evolvability could correspond to changes to an ancestral genetic system that permit greater evolutionary exploration (e.g., novel genes, changes to regulation, higher mutation rate), to the development of some ‘key phenotypic innovation’ that allows the descendant lineage to respond to diversifying selection, or to the opening up of some new environmental ‘niche’ that allows a variety of graded forms. Evolvability changes invite evolutionary speculation and hypothesis testing about phenotypes and genotypes, such as the suggested link between avian speciation rates and rates of molecular evolution^44^, cichlid speciation and genomic restructuring^38^ or intersexuality in moles^45^. As with the directional changes, they begin to help us understand the links between microevolution and macroevolution^46^.

We found no evidence to suggest that in the mammals at least, directional and evolvability changes catalysed each other, even though both are independently associated with size changes. This might simply reiterate that on a macroevolutionary timescale the two processes are distinct in their origins and causes. Nowhere, perhaps, is the separation of directional and evolvability changes more clearly illustrated than in the temporally early large-magnitude directional shifts. These look like the profile of an early burst but do not require or imply the changes to the Brownian variance that the early burst model expects^3^. These early changes, then, serve to remind us just how much a biased random walk with an unchanging Brownian variance can achieve.

The broad picture that emerges from our analysis is one of natural selection untethered from history in rates, directions and magnitudes of macroevolutionary change, not one structured by strong evolutionary trajectories or that requires special extra-Brownian jumps processes. The predominantly steady frequency and magnitude of changes when considered against the number of concurrent lineages would have pleased Darwin^46,47^. His principle of divergence envisaged a world in which interactions among species drive evolution more than historical contingency, climatic changes or other abiotic influences^46^. These species interactions yield a constant flow of new opportunities^48^, and can help to understand the steady pace of directional changes—towards becoming larger or smaller—and the steady pace of evolvability changes, towards increased and decreased ability to explore the trait-space. But, as might be expected from a constantly shifting biotic environment a strong bias emerges towards increased evolvability. If the mammals are representative, it might be time to reconsider notions of local optima beckoning (but see ref. 35 for a different view) and then ensnaring species: the pace of change and the ability of species to respond to it, might just render them ephemeral on a macroevolutionary timescale.

## Methods

### Model

Write the value of some trait *Y* after an amount of time *dt* as the outcome of multiplicative diffusion from its starting position at time *t* and a change term incorporating possible directional and evolvability effects:

1 $$Y\left(t+{dt}\right)=Y(t){e}^{\beta {dt}+\varepsilon (\upsilon ,{dt})}$$

where *β* is a directional change (per unit time), random changes *ε* are time-independent and homogeneous such that $\varepsilon \sim N\left(0,{\sigma (\upsilon ,{dt})}^{2}\right)$, and *υ* transforms the variance of *ε* to *υσ*^2^. These parameters correspond to the directional and evolvability changes as described in Fig. 1. By definition, directional changes occur along phylogenetic branches, introducing a mean offset to all ‘downstream’ species, but with no change to the Brownian variance. Evolvability effects occur at phylogenetic nodes, altering the Brownian variance *σ*^2^ of the descendant clade.

The model of Eq. (1) captures the commonly observed dependency in morphological data between a trait’s value, *Y*, and its variance. Writing the model in logarithmic form yields

2 $${Log}\left(Y\left(t+{dt}\right)\right)={Log}\left(Y\left(t\right)\right)+\beta {dt}+\varepsilon \left(\upsilon ,{dt}\right),$$

and Eq. (2) defines a linear model with constant and normally distributed errors *ε*, independent of the value of *Y*. For traits with variance independent of *Y* on the natural (un-transformed) scale, the model of Eq. (2) is still applicable, but *Y* is not logarithmically transformed.

Positive values of *β* denote increases in the value of the trait, negative values denote reductions. When the trait’s change along a branch is compatible with that which is likely under neutral drift, *β* = 0, its default value. The default value of *υ* is 1; values of *υ* > 1 signal an increase in the potential for evolutionary exploration, those <1 indicate reduced exploration of the trait-space. The existence and magnitude of directional and evolvability parameters that differ from their default values will vary throughout the tree according to the macroevolutionary signals retained in the species.

The model of Eq. (2), when applied to the branches leading from the root of the phylogenetic tree to the tips, yields a description of each species as the sum of the common ancestral state at the root, *α*, any directional changes that have occurred along the branches leading to that species, and a normally distributed error. Writing the *i*^*th*^ species’ value as *Y*_*i*_, and treating *Y*_*i*_ as having been logarithmically transformed where necessary, then

3 $${Y}_{i}=\alpha +{\beta }_{i1}{t}_{1}+\ldots {\beta }_{{ij}}{t}_{j}+{e(\Sigma {\upsilon }_{{ij}}{t}_{j})}_{i},$$

where the *β*_*ij*_ are directional changes occurring in the branches of length *t*_*j*_ (replacing the *dt* of Eq. (2)) leading to species *i*, and the error term has variance ${\sigma }^{2}\Sigma {\upsilon }_{j}{t}_{j}$.

If all ${\beta }_{i}=0$, and all ${\upsilon }_{j}=1$, the model simplifies to unbiased constant-variance Brownian motion. More generally, Eq. (3) predicts that species will have different expected values and variances, yielding a multivariate normal distribution of species’ trait values such that the ${Y}_{i} \sim N({{{{{\boldsymbol{u}}}}}}{{{{{\boldsymbol{,}}}}}}{{{{{\boldsymbol{\Sigma }}}}}})$. The mean vector ***u*** is calculated for each species from $\alpha +\mathop{\sum}\nolimits_{j}{\beta }_{j}{t}_{j}{{{{{\boldsymbol{,}}}}}}$ where the summation for species *i* is over the *j* branches along the path length leading from the root of the tree to species *i*. The matrix **Σ** is given by ***V***_***υ***_, the variance-covariance matrix among species as implied by the phylogeny given the assumption of Brownian motion, and any changes to evolvability, *υ*: a species’ variance is as given above $({\sigma }^{2}\Sigma {\upsilon }_{j}{t}_{j})$ and the covariance between any two species (tips of the tree) is proportional to their shared path length in the tree. The log-likelihood of this multivariate normal for the observed trait data at the tips of the phylogeny is

4 $${{\log }}{{{{{\rm{L}}}}}}\,\left({{{{{\boldsymbol{Y}}}}}}\right)\propto \mathop{\sum}\limits_{i}\left[{{\log }}\,\left({{{{{{\boldsymbol{Y}}}}}}}_{{{{{{\boldsymbol{i}}}}}}}\right)-\left(\mu +\mathop{\sum}\limits_{j}{\beta }_{{ij}}{t}_{j}\right)\right]^{\prime} {{{{{{{\boldsymbol{V}}}}}}}_{{{{{{\boldsymbol{\upsilon }}}}}}}}^{-1}\left[{{\log }}\,\left({{{{{{\boldsymbol{Y}}}}}}}_{i}\right)-\left(\mu +\mathop{\sum}\limits_{j}{\beta }_{{ij}}{t}_{j}\right)\right]$$

where ***Y*** is a vector corresponding to the trait data in the *n* species. For computational purposes, values of *υ* > 1 alter the Brownian variance by effectively lengthening phylogenetic branches, values of *υ* < 1 shorten them^5^; directional changes do not alter ***V***_***υ***_^49^. A consequence of this computational convenience is that a preponderance of *υ* > 1 will lead to a smaller overall estimate of *σ*^2^ for any fixed amount of variation among species, although this has no effect on any of the model’s other results.

The model of Eqs. (3) and (4) can also include a global directional trend parameter *β*_*g*_. The global directional parameter measures the slope of root-to-tip (species) directional change against variation in elapsed root-to-tip ‘time’. Elapsed time might vary in trees containing fossil species, or, as here, we estimate *β*_*g*_ using variance-adjusted time^4,5,49^, such as the *υ* bring about. Variance-adjusted time is the equivalent number of years of evolution at the background Brownian variance, and *β*_*g*_ tests whether a greater number of such years is associated with more phenotypic divergence from the ancestral state at the root of the tree (or vice versa). Either form (fossils or variance-adjusted years) raises the possibility of improving estimates of ancestral states and, unlike non-directional models, of estimating ancestral states that fall outside the range of observed values at the tips of the tree^4,49^.

Rewriting the model to include a global directional trend

5 $${{\log }}{{{{{\rm{L}}}}}}\,\left({{{{{\boldsymbol{Y}}}}}}\right)\propto \mathop{\sum}\limits_{i}\left[{{\log }}\,\left({{{{{{\boldsymbol{Y}}}}}}}_{{{{{{\boldsymbol{i}}}}}}}\right)-\left(\mu +\mathop{\sum}\limits_{i}{\beta }_{{ij}}{t}_{j}+{\beta }_{g}{T}_{i}(\upsilon )\right)\right]^{\prime} {{{{{{{\boldsymbol{V}}}}}}}_{{{{{{\boldsymbol{\upsilon }}}}}}}}^{-1}\left[{{\log }}\,\left(\left({{{{{{\boldsymbol{Y}}}}}}}_{{{{{{\boldsymbol{i}}}}}}}\right)\right)-\left(\mu +\mathop{\sum}\limits_{i}{\beta }_{{ij}}{t}_{j}+{\beta }_{g}{T}_{i}(\upsilon )\right)\right]$$

where *β*_*g*_ relates the trait data to the vector of adjusted time *T*(***υ***) that results from the application of the evolvability multipliers (*υ*) throughout the tree. For computational convenience, *T*(***υ***) is measured in units of variance-years as above: the equivalent number of years of evolution at the ‘background’ Brownian variance.

Let **Φ** be a vector of the parameters of the model of trait evolution including the nominal ancestral value, $\alpha$, the vector of directional change parameters, ***β***, the vector of evolvability parameters, ***υ***, and the global directional parameter ${\beta }_{g}$. These directional and evolvability parameters are initialised at their default values, and the ancestral *α* and global directional change parameters are initialised at the mean of their prior distributions. Then, we estimate the posterior density of **Φ** in a Bayesian Reversible-Jump Markov chain Monte Carlo (RJ-MCMC)^50^ setting that allows the number of directional and evolvability parameters that depart from their default values to vary around a stationary set from one iteration to the next. The posterior density is written as

6 $$P({{{\mathbf{\Phi }}}} {|}D)\propto \int L\left(D|{{{\mathbf{\Phi }}}} \right)p\left({{{\mathbf{\Phi }}}} \right)d({{{\mathbf{\Phi }}}} )$$

where $L\left(D|{{{\mathbf{\Phi }}}} \right)$ is given by Eqs. (4) or (5), the $p\left({{{\mathbf{\Phi }}}} \right)$ are the prior distributions of the parameters of the model of trait evolution, and the Monte Carlo integration is over $d({{{\mathbf{\Phi }}}} )$. We choose the $p\left({{{\mathbf{\Phi }}}} \right)$ to be compatible biologically with the processes they represent (Methods, Priors on model parameters).

### Model fitting and posterior data

We find the set of parameters in **Φ** using a RJ-MCMC procedure that we have employed elsewhere to search high-dimensional spaces^51,52^: *Add/Remove* proposals either attempt to add or remove $\beta ^{\prime} s$ or $\upsilon ^{\prime} s$ from the model by changing them from or returning them to their default values, respectively; *Change* proposals attempt to add or subtract a value to or from a *β* or *υ*, not at their default values. A value of *β* is always paired to the *t* or branch length of the branch in which it occurs to make $\beta \times t$, which is then assessed against the prior (below). Proposed changes to the model are accepted or rejected according to the usual Metropolis-Hastings algorithm (see below). At stationarity, the Markov chain yields posterior distributions of directional and evolvability parameters at each branch or node of the tree (most of the branches and nodes will never receive a parameter and will have values of these parameters set to one or zero, respectively).

We ran the Markov chain until it reached stationarity, drawing 1000 posterior samples from the stationary chain at widely spaced intervals to ensure a low (r ~ 0.1) autocorrelation among successive sampled iterations. The parameter values we report are means averaged over these 1000 samples, having adjusted the sample size for autocorrelation (see also Selection of parameters from the posterior distribution, below). We repeated this at least six times (see *Selection of parameters*).

### Model comparisons

We calculated marginal likelihoods for each model in Table 1 via a stepping-stone sampler implemented in *BayesTraits*, using 1000 stones, each run for 50,000 iterations. Marginal likelihoods numerically integrate the likelihood over the entire hyper-volume of the parameter space the priors define. This averaging thereby naturally penalises models with more parameters or models whose priors include regions in which the model fits badly–adding a parameter to a model can sometimes reduce its marginal likelihood. The difference in the marginal likelihoods among models can be treated as a *BayesFactor*.

### Priors on model parameters

We apply a Gamma prior to the evolvability changes

$$\upsilon \sim {{{{{\rm{Gamma}}}}}}(\alpha ,\beta )$$

where *α* = 1.2 and *β* = 5. This produces a right-skewed distribution with a lower limit of zero and a mode of 1, the default value. We use this prior over a uniform or other relatively unconstrained priors so as to improve the efficiency of the Monte Carlo search, but without constraining the posterior.

We assume a ‘star phylogeny’ (no phylogenetic structure) and then obtain a probability distribution on the evolvability parameters by noting that

$$\upsilon \sim \frac{{\hat{\sigma }}^{2}}{{\sigma }^{2}} \sim \frac{{\chi }^{2}}{n-1}$$

where $\frac{{\hat{\sigma }}^{2}}{{\sigma }^{2}}$ is the ratio of the observed variance (for example in some clade) to the Brownian variance, and *n* specifies the number of taxa that descend from the affected node. Then, within a clade that attracts a *υ*,

7 $$f\left(\upsilon \right)=\frac{{2}^{-n/2}n{e}^{\frac{-{nx}}{2}}{({nx})}^{\frac{n}{2}-1}}{\Gamma (\frac{n}{2})}$$

where Γ is the gamma function. The density $f\left(\upsilon \right)$ has *μ* = 1 and ${\sigma }^{2}$ = 2/n and for the special case of *n* = 2 simplifies to the ${\chi }_{1}^{2}$ distribution. We use $f\left(\upsilon \right)$ to estimate the upper and lower 5% cut-points of *υ* for any given number of descendant taxa *n*, which as predicted *(*e.g., Fig. 3d) grow wide for small *n*.

As a prior to the directional changes, we assume

$${{{{{\rm{|}}}}}}{{\beta }}\times t{{{{{\rm{|}}}}}}{{{{{\boldsymbol{\sim }}}}}}{Weibull}(\kappa ,\lambda ),$$

where *t* is the length of the branch in which the change occurs. For the analyses we report here we set *κ* = 1.5 (shape) and *λ* = 1.1 (scale), giving a right-skewed distribution with a lower limit of zero, a mode of ≈0.5 and upper range of ≈3. As with the υ, this distribution of directional changes emerged from many preliminary trials using uniform priors or other relatively unconstrained priors, and we use it to improve the efficiency of the Monte Carlo search. The range of posterior |$\beta \times t$| values it returns (e.g., Fig. 3a) can be shown to be compatible with expectations derived from estimates of within-population variances (Fig. 4, Supplementary Table 3).

Other priors might prove appropriate for different traits or for different taxonomic groups, and this is a minor research area in its own right; alternatively, one could derive a prior based on estimated within-population variances. We additionally charged 2 log units per directional parameter, equivalent to assuming that the prior probability of a directional effect occurring in a branch is ~1/7^th^. This adjustment made directional and evolvability parameters approximately equally costly despite their prior distributions having different probability densities.

We set a normal prior centred at 0.0 and with a standard deviation of 0.1 on the global directional parameter, and a Gamma prior $(\alpha =2,\beta =0.7)$ on the ancestral value at the root of the tree, and with a threshold of −3 to centre the prior at a value equivalent to 25 g. This produces a right-skewed distribution with a wide range (95% CI = 0–383 g). In none of the above cases does our choice of priors qualitatively affect our conclusions.

### Selection of parameters from the stationary Markov chain

At stationarity reversible jump Markov chains sample a fluctuating set of parameters around some stable core. Our interest is to study the model’s parameters for their properties, so we need a formal way to identify a ‘reliable’ set in the posterior sample as some will move in and out of the stationary chain at chance probabilities. We describe here how to identify those parameters whose posterior probabilities of being in the posterior sample exceed an amount expected by chance. An important feature of our selection process is that parameters are selected (or not) from the posterior sample for further study on the basis of their posterior frequency of occurrence in the chain, not on their magnitude.

Formally, the Metropolis-Hastings algorithm accepts changes to the model (i.e., parameters being added, removed or altered in magnitude) according to

$${{{{{\rm{P}}}}}}({{{{{\rm{acceptance}}}}}}\; {{{{{\rm{of}}}}}}\; {{{{{\rm{change}}}}}})={{\min}}\{1,\left(P(D|{{{{{{\mathbf{\Phi}}}}}}}^{{\prime} })p\left({{{{{{\mathbf{\Phi }}}}}}}^{{\prime} }\right)/P\left(D|{{{{{\mathbf{\Phi }}}}}}\right)p\left({{{{{\mathbf{\Phi }}}}}}\right)\right)\},$$

where the primes denote the model with proposed changes and *P*(*D*|**Φ**) is the probability of the data given the model of evolution. The Metropolis-Hastings algorithm accepts a parameter with probability = 1 if it improves the likelihood of the model, otherwise, changes are accepted with probability given by the ratio of the likelihood of the new model to the old model.

Let *p* be the prior probability of a parameter proposed to be added to the model, where *p* varies with the size of the parameter, as specified by its prior. Then, even if inclusion of the parameter does not alter *P*(*D*|**Φ**), the Metropolis-Hastings algorithm will accept this new parameter with probability *p*. Averaged over many iterations of a stationary chain, a parameter will achieve a posterior probability of ~$p^{\prime} \pm {(\frac{p^{\prime} \left(1-p^{\prime} \right)}{n(1-{r}^{2})})}^{1/2}$ where the prime on *p* denotes the observed posterior probability, *n* is the number of sampled iterations of the chain and *r* measures the autocorrelation among successive iterations.

We consider posterior probabilities that exceed the chance *p* by two standard deviations to have occurred significantly more often in the Markov chain than expected by chance, and to be candidates for inclusion in the posterior. Chance levels of *p* were calculated for each estimated parameter by calculating their prior probabilities in either the Gamma or Weibull densities, as appropriate, based on their mean value in the posterior sample. We estimated *p'* separately for each directional and evolvability parameter from its posterior probability of occurrence in the stationary Markov chain. We then found those parameters whose posterior probabilities exceeded the ‘chance’ cut-point by two standard deviations around *p'*.

We ran each Markov chain to stationarity six times. We chose for inclusion in the final set of posterior parameters those that exceeded the two standard deviation criterion described here in all six runs. This conforms to a two-tailed binomial probability of 0.032 under the assumption of a 0.5 chance of inclusion per run. Of the parameters that occurred in at least one posterior set, most (71% for both directional and evolvability) fulfilled this ‘six’ criterion; the remaining 29% appeared in roughly equal proportions in 1–5 of the posteriors. We find that this extra step of excluding parameters that appear in fewer than six runs removes parameters that might reflect idiosyncrasies of any particular Markov chain or parameters that hover around having ‘chance’ effects. This yielded *n* = 427 and *n* = 161 directional and evolvability changes, respectively.

Intercorrelations between the mean parameter values that made it into the final set averaged between 0.98 and 0.99 among the 15 pairs of runs. We then chose for use in all further analyses, the single run of the six whose parameter values had the highest average intercorrelation with the other five runs. We chose this approach in preference to averaging the six runs to retain the direct link between features of the parameters. It would have made no difference to our results to use the averages.

### Discriminating ‘trade-offs’ from linked effects

Among the 427 directional change parameters and 161 evolvability parameters, sixty pairs (*β*, *υ*) occur in tandem on the tree—one along a branch, the other on the node of the tree at the end of the same branch. These sixty pairs might be suggestive of linked or paired evolutionary effects. For each pair, we calculated the probability by chance that they would have occurred together, given their individual posterior probabilities of occurrence at those positions in the stationary Markov chain. Using a two standard deviation criterion, we found that fifty-two of the sixty occurred together significantly less often than expected by chance.

This result indicates that when one of the parameters was present in the stationary chain the other tended not to be, suggesting that rather than being linked the two effects were trading-off in the model. To test this, and to identify the more probable of the two effects, we examined their individual posterior probabilities of occurrence at their positions in the tree. Again, using a two standard deviation criterion, we found that in 42 cases the directional effect was significantly more probable than the evolvability effect; these were assigned to be directional effects. In *n* = 10 cases the evolvability effect was significantly more common than the directional effect and these were assigned to be changes to evolvability. In eight cases neither parameter occurred significantly more often than the other and these were retained in the posterior sample as tandem effects.

This step yields a final tally of 417 and 119 directional and evolvability parameters, respectively (the thinning procedure does not alter any of our qualitative results). With 5421 branches in the tree (the tree contains some polytomies) the number of directional effects compares to the 5% Hunt^53^ reports for fossil temporal sequences.

### Reporting summary

Further information on research design is available in the Nature Research Reporting Summary linked to this article.

## Supplementary information

Supplementary Information

Reporting Summary

## Data Availability

All species’ body mass data used here are previously published in references^24,25^. Source data are provided with this paper.

## Source data

Source Data

## Code availability

Analyses were run using the computer programme BayesTraits v.4 which is freely available from http://www.evolution.reading.ac.uk/BayesTraits.html.
