# Supplementary material for: General statistical model shows that macroevolutionary patterns and processes are consistent with Darwinian gradualism
Source: Nat Commun. 2022 Mar 2;13:1113. doi: 10.1038/s41467-022-28595-z (PMC8891346; doi:10.1038/s41467-022-28595-z)
Supplement: Supplementary file 1 — Supplementary Information [file 41467_2022_28595_MOESM1_ESM.pdf]

Supplementary Information

General statistical model shows that macroevolutionary patterns and processes are consistent with Darwinian gradualism

Mark Pagel, Ciara O'Donovan[1] and Andrew Meade[1]

School of Biological Sciences, University of Reading, Reading, RG6 6UR, UK.

Contact information:

Mark Pagel
School of Biological Sciences,
University of Reading, Reading, UK

Email: m.pagel@reading.ac.uk

Supplementary Methods

Simulation studies. We simulated Brownian motion on the phylogeny of Supplementary Figure 1 by drawing unit time incremental steps distributed ~*N(0,0.004)*, simultaneously adding directional and evolvability effects to a clade of 32 taxa (dark grey shading). We varied directional changes (β) in intervals of 0.1 from zero (no effect) to 5 (large positive effect) in the indicated (purple) branch. We did the same for changes to evolvability (υ) at the node marked with the green circle We varied the parameters in a fully-crossed design, yielding 2600 parameter combinations. Directional effects shift the mean phenotype of the dark grey clade (n=32 species) with no change to the Brownian variance, evolvability effects change the Brownian variance of the species within the grey clade, with no change to the mean phenotype.

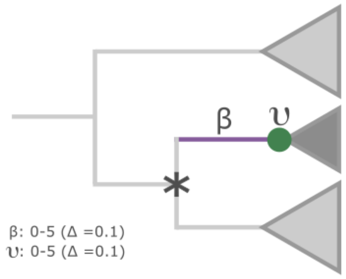

Supplementary Figure 1. Description of the simulation study.

The scenarios described in Supplementary Figure 1 pose a challenge to the Fabric model. For example, a directional change will alter the mean difference between the species in the dark grey clade and the species in the other two clades. This will increase the phenotypic variance between the dark grey clade and its sister clade (and even to some extent with the outgroup clade). The model might respond to this by increasing the Brownian variance at the node marked by the asterisk, rather than by putting a directional effect along the purple branch. That is, what could look like an increased variance from the perspective of the asterisk-node is actually a directional change that can be explained as a biased random walk with no change to the Brownian variance.

The signal that discriminates these two scenarios is the phenotypic variance within the dark grey clade: if it conforms to that expected from the Brownian variance, then the mean phenotypic change is almost certainly the result of a directional effect and not a change to evolvability. If the phenotypic variance within the dark grey clade is increased relative to that expected from the Brownian variance, then the apparent phenotypic change might be the result of a change in evolvability.

Similarly, a change to evolvability acting at the green circle could be mistaken for a directional change because altering the variance within the dark grey clade can, by chance, change its mean value relative to the other clades. In this case, the model might mistakenly place a directional effect along the purple branch leading to the dark grey clade.

We analysed each of the 2600 simulated datasets to see if the model could locate and separate the two kinds of effect. Supplementary Table 1 reports the correlations between the simulated and MCMC estimated parameter values across all 2600 parameter combinations, using the same MCMC model as described in the main text. The estimated directional effects closely tracked the simulated values, independently of any changes to evolvability affecting the descendant clade.

For the evolvability parameters, the correlation was slightly lower but again indicate that the estimated clade-wise phenotypic variances tracked the simulated values, independently of the mean offset of the clade owing to any directional effect.

Supplementary Table 1. Correlations and regressions of estimated directional ($\boldsymbol{\beta}$)and evolvability ($\boldsymbol{\upsilon}$) effects against the known value in a simulated dataset with a target clade of 32 taxa; n=2600 parameter combinations. Source data are provided as a Source Data file.

Parameter	correlation	slope	intercept	n
$\boldsymbol{\beta}$	0.977	0.959	-0.006	2600
$\boldsymbol{\upsilon}$	0.836	0.925	0.253	2600

In a multiple regression including both simulated effects and their interaction, and used to predict either the estimated directional effects (R = 0.977) or the estimated evolvability multipliers (R = 0.836), the correlation is unchanged (to three decimal places) over the simple bivariate correlations, and only the relevant main effect was significant in each case.

In a separate set of simulations, we inserted 37 directional and 11 evolvability changes in random places on a background of Brownian motion using the Carnivore clade from the Time Tree of Life (n=257 species). Thirty-seven directional effects and 11 evolvability changes on a tree of this size mimics the ratio of tips to parameters, and their division into the two classes, that we found in the mammal tree. We repeated this 1000 times randomly varying the position in the tree and the magnitudes of the directional and evolvability changes from one simulation to the next, drawing values of both sets of parameters randomly from the priors we used for the analysis model reported in the main text. We used a Brownian variance of 0.0031, similar to that we found across all mammals (see Table 1, main text). Random directional shifts drawn from the prior were randomly assigned to be positive or negative.

The 1000 independent sets of simulated data each contained information on 48 independent parameters -- 37 directional and 11 evolvability changes. We analysed each set of data with the Fabric model using the same settings as we used for the mammal data, running each Markov chain to stationarity before sampling 1000 iterations for the posterior sample, and estimated effects as the average of their values over this posterior. The model did not know *a priori* the location or value of the parameters.

The correlations we report in Supplementary Table 2 analyse the value of the parameter the model estimated at the correct position in the tree against the true value of the parameter at that position. The directional changes are well estimated across the range of their prior and in the presence of other directional and evolvability changes. Confining the analysis to estimated directional effects with coefficients of variation < 0.5, indicative of effects that left strong signals in the simulated downstream data, the correlation improves slightly.

Supplementary Table 2. Correlations and regressions of estimated directional ($\boldsymbol{\beta}$)and evolvability ($\boldsymbol{\upsilon}$) effects against the known values in 1000 simulated datasets with multiple simultaneous effects[a]. Source data are provided as a Source Data file.

	**Overall sample**			**Subset sample[b,c]**		
Parameter	correlation	slope	n	correlation	slope	n
$\boldsymbol{\beta}$	0.95	0.93	37000	0.97	0.97	29212[b]
$\boldsymbol{\upsilon}$	0.63	0.58	11000	0.79	0.96	2308[c]

a) the 37 directional and 11 evolvability changes on a tree of 257 species matches the density and division of parameters found on the full mammal tree; the analysis model is blind to the location and magnitude of the simulated effects; b) estimated directional and evolvability parameters with coefficients of variation in the posterior sample of < 0.5; c) sample restricted to affected clade sizes of 10 or more species.

The evolvability effects are less well estimated (Supplementary Table 2) but the lower correlation between the estimated and true values is still highly significant. This lower correlation is expected from the probability density of the υ (eq. 7, Methods, main text and dashed curves Figure 3d), whose

variance increases steeply as the number of affected taxa gets smaller. Confining the analysis to estimated υ that affect 10 or more taxa, the correlation and slope improve considerably.

Identifiability. When two or more alternative sets of parameters in a statistical model predict identical probability distributions of outcomes, the model cannot find a unique set of parameters to explain the outcome or response variable and the model becomes non-identifiable.

It is useful for studying identifiability of the Fabric model to express it as a multiple linear regression. Let ***X*** be a $n \times k + 1$ design matrix, where the *n* rows of ***X*** correspond to the *n* species in the phylogeny and the *k+1* columns correspond to a column of 1s, followed by *k* additional columns of dummy codes. The dummy codes correspond to the number of directional effects in the model.

A directional effect falling anywhere in the tree affects all of its downstream descendants' (as defined by the phylogeny) trait values by an amount $\beta \times t$ (see main text). Let β_k be the *kth* directional effect (the ordering of these *k* effects is arbitrary). Its dummy code in the matrix ***X*** will then have a '1' for each of these downstream species, and a '0' for all others.

With ***X*** defined this way we can write the model of equation 3 (main text) as:

$$\boldsymbol{Y} = \boldsymbol{X\beta} + \boldsymbol{e},$$

where ***Y*** is now a vector of the *n* species' trait values, ***X*** is the design matrix, $\boldsymbol{\beta}$ is the vector of *k* directional effects, and ***e*** is a vector of normally distributed random errors as defined in the main text (equation 3). Then,

$$\boldsymbol{\beta} = \left(\boldsymbol{X}'\boldsymbol{V}^{-1}\boldsymbol{X}\right)^{-1}(\boldsymbol{X}'\boldsymbol{V}^{-1}\boldsymbol{Y})$$

is the usual ordinary least squares estimator of the directional effects, where ***V*** is the variance-covariance matrix as given by the phylogeny under the assumption of Brownian motion, including any changes to the variance as a result of evolvability scalars (main text). Where ***V*** takes the form of an identity matrix scaled by a common variance – as in most non-phylogenetic settings – the equation for $\boldsymbol{\beta}$ simplifies to the familiar $\boldsymbol{\beta} = (\boldsymbol{X}'\boldsymbol{X})^{-1}(\boldsymbol{X}'\boldsymbol{Y})$. For simplicity, the vector $\boldsymbol{\beta}$ is here equivalent to a vector of individual $\beta \times t$ such as we report in the main text. To retrieve the individual β on their own, the dummy codes in the design matrix ***X*** must be scaled by the appropriate branch lengths.

Least squares regression models are identifiable so long as $\left(\boldsymbol{X}'\boldsymbol{V}^{-1}\boldsymbol{X}\right)^{-1}$ has an inverse (non-singular matrix). We used this property to study the identifiability of the Fabric model for the mammal dataset used in this paper, and in simulated data. In the current context, the design matrix ***X*** is not known in advance. Rather, Markov chain Monte Carlo can be seen as a methodology for discovering design matrices that, in this instance, retrieve historical effects, something our simulations confirm. At stationarity, the Markov chain yields a set of parameters that fluctuate around some average or stationary number. We ran six separate Fabric model replicates (the combined model of Table 1, main text) to stationarity on the mammal data, sampling 1000 widely-spaced iterations of the model from the end of the stationary chain, constructing ***X*** matrices for each iteration. We found that on average 91.7% of the design matrices yielded identifiable models, ranging from 91.5 to 92.8 across the six independent chains.

We then generated 1000 sets of 37 directional effects and 11 evolvability effects, placing the effects in random locations on the Carnivore clade of the Time Tree of Life (as used for the simulations in Supplementary Table 2). As with the previous simulations, this number of parameters mimics the density of directional and evolvability parameters we found in the mammal tree. On average 90.1%

(Supplementary Figure 2a) of these random design matrices were identifiable, suggesting that non-identifiable sets of parameters arise by chance approximately 10% of the time for densities of effects such as we observe (Supplementary Figure 2a).

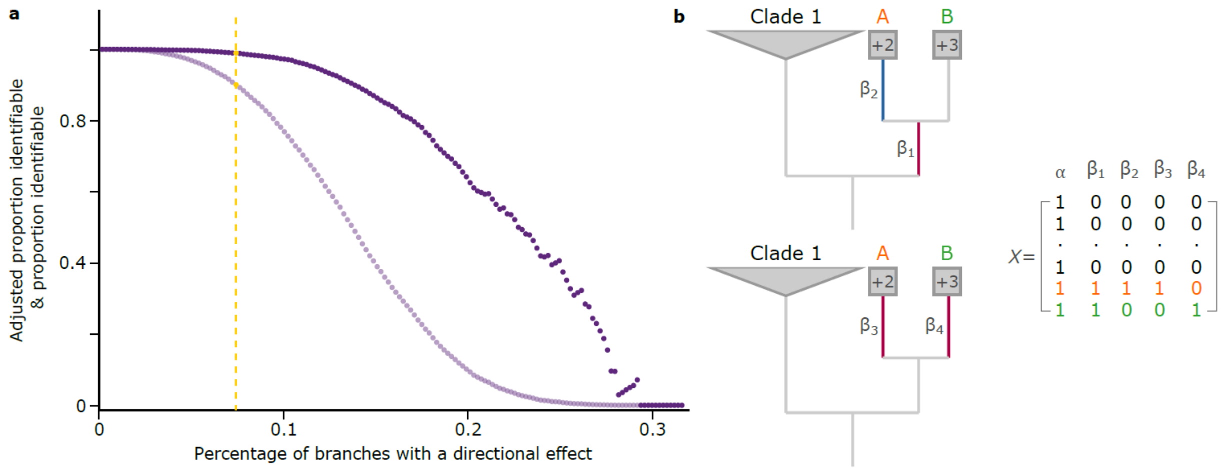

Supplementary Figure 2. Identifiability. **a**. Lilac curve: plot of the percentage of X'X matrices that are invertible against the proportion of branches with a directional effect. Varying numbers of directional effects randomly drawn from their prior were placed in random locations on the Carnivore clade of the Time Tree of Life. Each plotted point corresponds to 1000 random simulations with the proportion of directional effects as shown on the horizontal axis. The point corresponding to the density of directional effects we found for the mammal data is labelled (dashed yellow line). For these simulations the number of evolvability scalars was held constant at 11, but this doesn't affect the shape of these curves as the two sets of parameters can be added, here yielding 48 parameters. Dark purple curve: same relationship but having deleted from the sets of 1000 replicates instances in which the scenario in Supplementary Figure 2b arises (see also 2b text). **b**. Hypothetical and simplified example of non-identifiability that confronts all models of macroevolution concerned with trait evolution. Assume the species in clade 1, and the individual species A and B have trait values drawn from the same statistical distribution such that they differ only by random variation, save for the directional differences indicated in species A and B. Assume all branches are of length = 1. The design matrix *X* codes for two alternative sets of directional change parameters, $\{\beta_1, \beta_2\}$ and $\{\beta_3, \beta_4\}$ that predict identical outcomes in species A and B (coloured rows of X matrix). The set $\{\beta_1, \beta_2\}$ envisions a directional change leading to the common ancestor of species A and B, followed by a slight reduction along the branch leading to species A. The set $\{\beta_3, \beta_4\}$ describes independent changes leading to species A and B from a common ancestral value. Without further information as to the value of the trait in the common ancestor to species A and B, these two explanations of the species' values produce identical outcomes, and the matrix $(X'V^{-1}X)^{-1}$ does not have an inverse (there is an exact linear dependency between β_2 and β_3). In this hypothetical case, the set of parameters $\{\beta_1, \beta_2\}$ is more likely given the prior beliefs about the distribution of directional effects (see Methods, main text) and the MCMC will favour it over the set $\{\beta_3, \beta_4\}$. If instead of species A and B both differing from the expected distribution, only one of them does, then only a single change would be needed along the branch leading to that species and the parameters would become identifiable. Source data are provided as a Source Data file.

Nearly all cases of non-identifiability take the form of a problem in ancestral state or ancestral evolutionary process (e.g., directional change, evolvability change) reconstruction that all macroevolutionary-comparative models must confront. Two hypothetical species A and B in Supplementary Figure 2b have trait values arbitrarily set at +2 and +3 units larger than expected under a simple Brownian model. The parameter set $\{\beta_1, \beta_2\}$ expresses the belief the trait had increased by the time of their common ancestor, followed by a slight reduction in the branch leading to species 2 (assume all branches are of length = 1). The parameter set $\{\beta_3, \beta_4\}$ suggests the two increases in the value of the trait occurred independently in the two final branches. These two sets of parameters, $\{\beta_1, \beta_2\}$ and $\{\beta_3, \beta_4\}$, yield identical predictions, and are formally non-identifiable (Supplementary Figure 2b). Other values could be assigned to these two sets of parameters that will yield equivalent outcomes, but all such sets will by definition be non-identifiable (if instead of species A and B both having increased values, only one of them does, then only a single change would be required along the branch leading to that species and the model would become identifiable).

Without outside or extra information about the value of the trait in the past, deciding which set of changes is more likely requires assumptions about how evolution progresses. Parsimony and minimum evolution methods algorithmically resolve questions of identifiability by favouring scenarios that require fewer changes or less evolution. Here, a minimum evolution method would assign an intermediate value to the common ancestor followed by an increase in one species and a decrease in the other. The Fabric model decides between the scenarios in Supplementary Figure 2b by asking which is more likely given the prior assumptions the model makes about the distribution of directional effect sizes. The set $\{\beta_1, \beta_2\}$, requiring one large change and one small change is more probable than the two intermediate-sized changes of $\{\beta_3, \beta_4\}$ and the MCMC will favour it.

Approximately half of the branches of a phylogeny lead to species, making this hypothetical scenario likely when effects are placed on a tree at random locations. Deleting from the sets of 1000 replicates cases in which this scenario occurred yields the upper curve in Supplementary Figure 2a; now nearly all cases are identifiable within the density of directional effects we find.

Fossil evidence for abrupt and early large-magnitude changes. Alroy's[1] database of fossil mammals records n=1109 pairs of putative ancestor-descendant estimated body sizes, and the length of time separating the two fossils. We calculated the absolute change in $\log_{10}$(mass) for each pair, and normalised them by calculating an expected phenotypic variance using the Brownian variance from the directional change model (σ^2= 0.004, main text, Table 1) and the elapsed time separating the two fossils. We chose those pairs with z-scores > 2, as changes that might be indicative of a strong directional effect. This yielded n=588 pairs of changes (note: restricting the sampled pairs to the upper 10% of the z-scores yields 59 pairs and gives the same qualitative results as shown in Supplementary Figure 3).

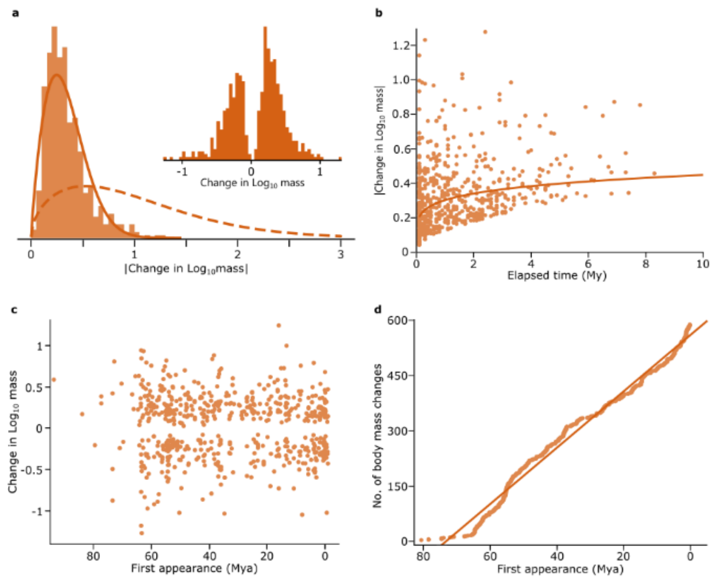

Supplementary Figure 3. **Changes in size in ancestral-descendant pairs of mammalian fossils**. **a** The absolute change in $\log_{10}$size for 588 ancestral-descendant pairs of mammalian fossils. The dashed curve is the prior used on the directional effects in the mammal data (main text), and the solid curve is a best-fitting log-normal; **b** Changes plotted against the length of time over which the change was measured (data from Alroy[1]). The curved lower limit of absolute change versus time indicates that changes of that magnitude or less fail to surpass the two-standard deviation criterion to be included as a directional change given their elapsed time; **c** Same data plotted against time of first appearance, appearing to show that a disproportionate number of large-magnitude changes occurred early in mammalian evolution, and smaller changes closer to the present, paralleling Figures 5a,b (main text); **d** After an initially slow period, directional effects accumulate broadly linearly with time (r^2 = 0.958), in contrast to Figure 6 (main text) possibly reflecting idiosyncrasies of this fossil dataset of ancestral-descendant pairs. Source data are provided as a Source Data file.

The histogram of absolute $\log_{10}$ changes for these 588 pairs (Supplementary Figure 3a) resembles that of Figure 3a (main text), albeit with a smaller mean. The absolute changes are relatively independent of time (Supplementary Figure 3b), similar to Figure 3b (main text). Elapsed times for the fossil data are approximately 1/10th of those in the mammal tree, and many of the largest changes occur over the shortest time spans. This supports the idea that the changes inferred from the model (see main text) occur over far shorter time periods than the length of the branch on the phylogenetic tree implies, and that the relationship in Figure 3b (main text) is not an artefact of long phylogenetic branches that might conceal extinction events.

Figure 5b (main text) shows that the size of directional phenotypic changes $(i.e., \beta \times t)$ the model detects early in mammalian evolution are large (absolute value) and decline closer to the present (slope=-0.0106, $p<0.0001$, n=417). We questioned whether that relationship is a limitation of the comparative method: perhaps only strong signals from the distant past will survive in the contemporary data.

Two lines of evidence are informative about this question. One is that the fossil data also show this decline (Supplementary Figure 3c), although with a shallower slope (slope=-0.0012, $p<0.0009$, n=588), leaving open the possibility that at least part of the decline in the reconstructed directional effects arises because small effects from the distant past get erased by later events.

The simulated data that we reported in the previous section provide a second line of evidence. We find that across all values of the simulated directional effects, the slope of the estimated effect against time since the common ancestor of the tree is not different from zero: slope = 0.0004988 (p=0.41). On the other hand, restricting ourselves to branches that have coefficients of variation < 0.5 for their associated b, the slope is -0.0061 ($p<0.0001$). This suggests that investigators should be aware that the declining size of phenotypic changes such as we observed in Figure 5b (main text) could be influenced by a limitation of the comparative method.

Magnitude of directional changes is compatible with a biased random walk. Under the null model of no directional effects in trait evolution, the expected amount of change that occurs along branches is zero, with a variance equal to $\sigma^2 t$, where σ^2 is the Brownian variance and t is the length of the branch in which they are found. Converting the variances ($\sigma^2 t$) to standard deviations yields changes in units of the trait (here, $\log_{10}$ body mass). The red (lower) curve in Supplementary Figure 4 plots the size of a two-standard-deviation unbiased random walk for a given branch length, and assuming a Brownian variance of σ^2= 0.004 per million years.

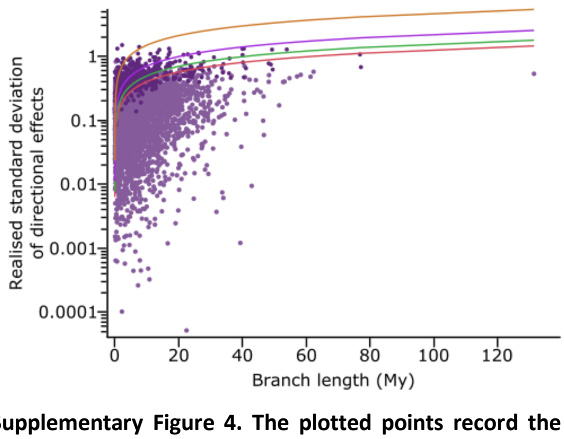

Supplementary Figure 4. The plotted points record the standard deviations of the branch-wise phenotypic directional changes, derived from Supplementary equation 2. Dark purple points are the 417 directional changes (main text), light purple are the remaining branches. Red curve: the upper limit of a two standard-deviation random walk for a given branch length, in units of trait change, setting σ^2= 0.004. Green, magenta and tan curves: the upper limits of *biased* random walks, in units of trait change, for biases equivalent to 1, 2 or 5 standard deviations of change per million years, respectively (Supplementary

equation 1). Nearly all dark purple points exceed an unbiased random walk, but fall inside five standard deviations per million years, or ~ 10-5 standard deviations or less per generation (see below). Source data are provided as a Source Data file.

Now assume that at every step of the random walk, there is a bias that changes some trait value x directionally by an arbitrary amount c per unit time. The variance of the difference in the trait value (σ_d^2) at succesive time periods *t* and *t+1*, is

$$\sigma_d^2 = \frac{(x_{t_i} - (x_{t_i+1} + c))^2}{2} = \frac{(x_{t_i} - x_{t_i+1})^2}{2} + \frac{c^2}{2} - c(x_{t_i} - x_{t_i+1})$$

When summed over many $t_i + 1 - t_i$ segments in a branch of total length t, the first term on the right above is just the variance of incremental Brownian motion, $\sigma^2 t$, the second term records the contribution to the variance of the many individual displacements c, and the third term has an expectation of zero because the individual displacements of the random walk are unbiased and centred around zero. The variance of the trait undergoing this biased walk over longer time period t is then

$$\mathrm{E}[\sigma_\mathrm{d}^2] = \sigma^2 t + \frac{\mathrm{c}^2}{2} t \qquad 1$$

The green, purple and tan curves in Supplementary Figure 4 correspond to cases of a two-standard deviation random walk, but where *ct* takes the value of one, two or five standard deviations of the variance, that is, the bias had led to the trait changing by one, two or five standard deviations over the length of the branch, on top of any variance of change from the random walk.

Equation S1 can be used to calculate the variances of change in the trait values brought about by the directional parameters, where βt now takes the role of c. Because the βt record the change along the length of the entire branch, as inferred from the actual differences among the species at the tips of the phylogeny, they include the variance of the random walk. Thus, σ_d^2 for these inferred changes is

$$\sigma_{d(\beta)}^2 = \frac{(\beta \mathrm{t})^2}{2}, \qquad 2$$

The plotted points in Supplementary Figure 4 record the standard deviations of the directional changes inferred along each branch of the Mammal tree, as derived from the Fabric model. Dark-purple dots are the n=417 directional changes in the posterior sample, lighter-coloured dots are changes that the model declared to be compatible with an unbiased Brownian random walk.

Nearly all the 417 observed directional changes fall above the red two-standard deviation unbiased Brownian random walk curve and below the biased random walk corresponding to a five standard deviation change per million years. We want to know whether these directional effects require any special evolutionary explanation. Previous reviews of field studies of wild populations suggest that three to five standard deviation changes to morphological traits can be achieved in as few as 50 generations[2,3]. But a non-trivial amount of the phenotypic variance that selection has acted on in these field population studies will have already existed in the study populations, and so they do not address the question of whether the supply of new mutational variance that enters the population each generation is sufficient to fuel directional selection occuring over the many millions of years represented by some of the branch lengths of a phylogenetic tree.

The critical question is whether typical within-population phenotypic variances, σ_w^2, are large enough to support sufficient amounts of per generation genetic change over long time periods to produce effects of the sizes observed in Supplementary Figure 4. The average within-population phenotypic variance in body size for twenty-three mammal species in one study[4] was 0.010 (log-transformed data) or 0.005 removing outliers (Supplementary Table 2). In a study of sixteen bird species[5] which reported the

variance of the morphological trait with the largest within-population variance for each species, the value was 0.011 (log-transformed data, excluding two-outliers). Estimated variances using log-transformed snout-vent length measurements in nineteen rattlesnake populations (*Crotalus viridis*) average 0.002 for females, and 0.003 for males[6]; including both sexes the average variance is 0.004.

These observed within-population variances are roughly comparable to the estimated Brownian variance per million years in this study, illustrating the slow realised pace of Mammalian morphological evolution. The per generation mutational variance component of the within-population variance is on the order of 10^{-2} to 10^{-4} of the environmental variance, which itself can be estimated at one-half or more of the phenotypic variance[7,8]. Using these values as guides, the within-population mutational variances σ_m^2 estimated from the phenotypic variances reported above for mammals are on the order of $\sim 2.5 \times 10^{-5}$ to 10^{-7} (Supplementary Table 2)

The median of the n=417 variances of change per million years calculated from supplementary equation 2 for the directional changes in the mammals, is $\sigma^2_{d(\beta)}/t = 0.036\ (95\%\ range\ 0.007 - 0.889)$. Adopting a conservative assumption of around 250,000 generations per million years for the average mammal (many mammals will have a million or more generations in this time), the 0.036 figure translates to a per generation variance of change of $\leq 1.5 \times 10^{-7}$ (Supplementary Table 3). This falls at the smaller end of the range of mutational variances, and is consistent with previous work comparing between and within-species variances[5,7]. The figure for the changes the model deemed compatible with neutral drift (light purple dots in Supplementary Figure 4 is $\sim 1.6 \times 10^{-8}$, an order of magnitude smaller.

Supplementary Table 3. Estimates of components of variance

'Typical' within population mammalian phenotypic variance = $\boldsymbol{\sigma_w^2}$	$\boldsymbol{\sigma_m^2}$ = estimate of per generation mutational variance component of $\boldsymbol{\sigma_w^2}$	Median per generation variance of change along a branch = $\boldsymbol{\sigma^2_{\Delta/g}}$
$\sim \mathbf{5} \times \mathbf{10^{-3}}$	$\sim 2.5 \times 10^{-5} \rightarrow 10^{-7}$	$\leq 1.5 \times 10^{-7}$

Note: all values are approximate.

This analysis, adapting a methodology developed by Lynch[7], and using conservative assumptions, points to the mutational variance being an adequate source of new variation to sustain long-term directional selection. It yields the expectation that the directional shifts we observe in the mammals are explicable as biased random walks against a constant-variance background, agreeing with previous work on this question[7,9]; indeed the range of directional phenotypic changes we observe is compatible with neutral drift[7,9]. Continued work on estimating within-population variances can usefully refine these conclusions. It might even be possible to use the estimated within-population mutational variance to define a prior on the directional effects in the Markov chain.

The plotted points in Figure 4 (main text) compare the values derived from Supplementary equation 2 for each directional change on the posterior sample, assuming an average generation time of four years, to $\sigma_m^2 * t * 250000$ where the product of branch length and 250000 is a conservative estimate of the number of generations in a branch of length t (measured in millions of years), and we use the smaller (10^{-7}) figure for σ_m^2.

Six early evolvability changes are not part of an evolutionary trajectory. Supplementary Figure 5 shows the six earliest changes to evolvability on the tree (main text, Figure 2). Red dots identify the locations of increased evolvability ($\upsilon > 1$, an increase to the Brownian variance), blue dots are the reverse ($\upsilon < 1$). The dotted circular-line identifies the approximate position of the K-T extinction event. Although one evolvability effect < 1 is nested within an earlier event of increased evolvability, it is not part of a general trajectory of a narrowing of the evolutionary variance in the mammals. The other two blue dots occur

after the two early red dots but are not descendant taxa. Even if these changes were part of a general evolutionary trajectory, this is opposite to the pattern expected from an OU process. That process expects that the realised differences among species are small early on, gradually rising to an equilibrium value. For later evolvability changes (see Figures 2, 4 main text).

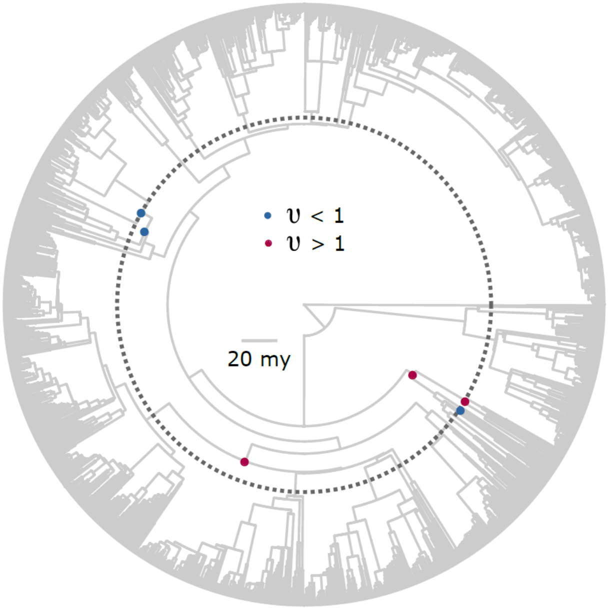

Supplementary Figure 5. The mammalian tree used in this study. The position and timing of the first six evolvability multipliers, showing that the progression from enhanced (red) to reduced (blue) evolvability is unlikely to be part of a trend because they are only partly nested. Dashed line is the K-T boundary.

Supplementary References

1 Alroy, J. Cope's rule and the dynamics of body mass evolution in North American fossil mammals. *Science* **280**, 731-734 (1998).

2 Hendry, A. P. & Kinnison, M. T. Perspective: the pace of modern life: measuring rates of contemporary microevolution. *Evolution* **53**, 1637-1653 (1999).

3 Kingsolver, J. G. & Pfennig, D. W. Patterns and power of phenotypic selection in nature. *Bioscience* **57**, 561-572 (2007).

4 Pagel, M. D. & Harvey, P. H. The taxon-level problem in the evolution of mammalian brain size: facts and artifacts. *The American Naturalist* **132**, 344-359 (1988).

5 Björklund, M. Processes generating macroevolutionary patterns of morphological variation in birds: a simulation study. *Journal of Evolutionary Biology* **7**, 727-742 (1994).

6 Ashton, K. G. Body size variation among mainland populations of the western rattlesnake (Crotalus viridis). *Evolution* **55**, 2523-2533 (2001).

7 Lynch, M. The rate of morphological evolution in mammals from the standpoint of the neutral expectation. *The American Naturalist* **136**, 727-741 (1990).

8 Clayton, G. & Robertson, A. Mutation and Quantitative Variation. *The American Naturalist* **89**, 151-158 (1955).

9 Lande, R. Natural selection and random genetic drift in phenotypic evolution. *Evolution* **30**, 314-334 (1976).
